# The effects of valproic acid on skin healing: experimental study in rats

**DOI:** 10.1590/acb370403

**Published:** 2022-07-15

**Authors:** Rachel Biondo-Simões, Maria de Lourdes Pessole Biondo-Simões, Sérgio Ossamu Ioshii, Rogério Ribeiro Robes, Moacir de Oliveira Dall’Antonia

**Affiliations:** 1 Fellow Master degree. Universidade Federal do Paraná – Postgraduate Program in Surgery Clinical – Curitiba (PR), Brazil.; 2 Full Professor. Universidade Federal do Paraná – Department of Surgery – Curitiba (PR), Brazil.; 3 Full Professor. Universidade Federal do Paraná – Department of Pathology – Curitiba (PR), Brazil; 4 Full Professor. Pontificia Universidade Católica do Paraná – Curitiba (PR), Brazil.; 5 Physician. Universidade Federal do Paraná – Veterinary Hospital – Curitiba (PR), Brazil.

**Keywords:** Valproic Acid, Epigenesis, Genetic, Wound Healing, Cell Proliferation, Collagen

## Abstract

**Purpose:**

To recognize the effects of valproic acid (VPA), an epigenetic drug, on the skin healing process.

**Methods:**

Sixty male Wistar rats were divided into two groups: the experiment treated with VPA (100 mg/kg/day); and the control, with 0.9% sodium chloride by gavage. Skin healing was studied in three moments (the third, the seventh, and the 14th day), evaluating the parameters: inflammatory reaction and its intensity (anti-LCA), angiogenesis (anti-CD34), collagen I and III (anti-collagen I, anti-collagen III and Picrosirius-red F3BA) and myofibroblasts (anti-alpha-AMS).

**Results:**

The inflammatory reaction was acute or sub-acute in both groups on the third day. On the seventh and the 14th day, chronic predominated in the control (p=0.006), and sub-acute in the experiment (p=0.020). There was a greater number of leukocytes in the group treated only on the third day (p=0.036). The number of vessels was lower in the treated group at the three times (p3=0.002, p7<0.001, and p14=0.027). Myofibroblasts were rare in the third day and moderate quantity in the remaining periods. Collagen I density was higher in the control at the three times (p<0.001) and collagen III in the treated group (p<0.001).

**Conclusions:**

VPA led to a more intense inflammatory reaction, decreased angiogenesis and collagen deposition, especially type I collagen.

## Introduction

Valproic acid (VPA), an established drug for the treatment of epileptic seizures and mania in bipolar disorder, has emerged as a promising drug in the treatment of tumors. It has proved to have potent antitumor effects both *in vitro* and *in vivo*, with encouraging results in early clinical trials, alone or in combination with demethylation and/or cytotoxic agents. Preclinical and preliminary clinical data strongly suggest that VPA could be a drug used a combination therapy, with classic cytotoxic drugs, with other molecularly targeted drugs or radiation, in many of solid tumors^1^. It was possible to verify that VPA alters proliferation, survival, cell migration, and hormone receptor expression of breast cancer cells both preclinically and clinically, and it can be used as an adjuvant agent in combination with many cytotoxic, hormonal and immunotherapeutic agents^2^.

Few studies have reported the influence effect of VPA on skin healing, with controversial results. Tseng et al.^3^ reported a patient whose traumatic ulcer only healed after sodium valproate was suspended. They argued that one of the side effects of VPA is hyperammonemia, which has been shown to impair platelet aggregation, in addition to thrombocytopenia^3^. Michaelis et al.^4^ adopted a negative view of VPA regarding the healing of wounds. According to these authors, collagen-stimulated platelet aggregation is lower in patients with schizophrenia treated with VPA. They reported reduced activity of the arachidonate cascade in platelets, inhibiting thromboxane A2 synthesis, impairing adenosine triphosphate release and platelet aggregation.

Lee et al.^5^ studied, in a murine model, the topical application of VPA to a full-thickness wound. After seven days, they observed marked healing and reduction in wound size in mice treated with VPA. They reported greater migration of keratinocytes, suppression of apoptosis and greater concentration of types I and III collagen. Thus, for these authors, at the cutaneous level there was an improvement in healing. Bambakidis et al.^6^ studied VPA in pigs and reported increased fibrinolysis. Darby and Hewitson^7^ observed that the levels of α-SMA, which identifies contractile proteins and therefore myofibroblasts, as well as markers for collagen I and collagen III were increased in wounds treated with VPA. Ala et al.^8^ used valproate at different dosages, in rats, intraperitoneally, and reported that skin flaps healed faster in the VPA group.

It has been shown that, in bladder tumor cell lines, VPA dose-dependently decreased cell proliferation and DNA synthesis and induced G1 phase blockade^9^. Furthermore, *in-vitro* and *in-vivo* antiangiogenic effects of VPA have been identified^10^. These effects are associated with the downregulation of pro-angiogenic genes, such as vascular endothelial growth factor (VEGF) and/or endothelial nitric oxide synthase (eNOS) genes. In addition, histone deacetylation (HDAC) inhibitors induce hyperacetylation of HIF-1α, a pro-angiogenic transcription factor, which induces its degradation^11^.

Carcinogenesis is a cumulative and microevolutionary process that results in uncontrolled cell proliferation and the invasive phenotype^12^. It cannot be explained by genetic alterations alone, but also involves epigenetic processes^11^.

Epigenetic alterations are defined as inherited modifications that are not present in the DNA sequence. Gene expression is regulated at many levels and not only in response to changes in DNA. Examples of epigenetic control are DNA methylation, histone deacetylation and mi-RNA expression. Methylation of several tumor suppressor gene promoters is responsible for their silencing and therefore potentially supports carcinogenesis. Likewise, histone deacetylation can lead to oncogene activation. Mi-RNAs are small non-coding RNA fragments (18-20 nucleotides) capable of inhibiting other m-RNAs, ultimately altering the balance in oncogene gene expression and tumor suppressor. Drugs capable of interfering with these mechanisms may have a positive impact on tumor progression. Indeed, epigenetic changes are dynamic and can be reversed by epigenetic inhibitors. Recently, methyltransferase and histone deacetylase inhibitors have attracted the attention of researchers and clinicians as they potentially provide therapeutic alternatives for some types of cancer^12^.

It has been proved that several epigenetic drugs prolong the survival of patients and are less toxic than conventional chemotherapy^13^. They have been used to treat several types of cancer, such as hematological^14^, breast neoplasms^15^,^16^, cancer of the bladder^17^, lung cancer^18^,^19^, ovarian cancer^20^, prostate cancer^21^, colorectal cancer^22^, and hepatocellular carcinoma^23^.

Among the drugs approved by the Food and Drug Administration (FDA) that inhibit DNA methyltransferases, there are azacitidine and decitabine, and among those that inhibit histone deacetylases, vorinostat and romidepsin. These early successes demonstrate the potential of epigenome-targeted therapies^24^.

If we consider that, in the treatment of tumors, HDAC inhibitors, including VPA, have been shown to be capable of altering cell proliferation and migration^1^,^2^,^9^, promote thrombocytopenia^3^,^25^,^26^, decrease fibrinogen and VEGF levels^27^, and it is to be expected that they can affect the healing process.

Since VPA is a well-known and low-cost drug, there is a possibility that it can be used as a neoadjuvant drug in association with other chemotherapeutics, and that all these drugs interfere with the healing process, it is important to recognize whether this association would have the potential to weaken the process.

Thus, the aim of this study was to recognize, in a murine model, the effects of VPA on the skin healing process by analyzing the inflammatory reaction, angiogenesis and collagen synthesis.

## Methods

This study was conducted following evaluation and approval by the Committee of Ethics for the Use of Animals in the Biological Science Sector (CEUA-Biológicas) of the Universidade Federal do Paraná (UFPR), registered as number 1313, process 23075.058610/2019-42. Approval was given on June 18, 2019. The study was conducted in compliance with Federal Law 11,794 of October 8, 2008 and followed the guidelines of the Brazilian society of Science in Laboratory animals (COBEA).

### Sample and division of groups

The study was conducted with 60 male Wistar rats (*Rattus norvegicus albinus*, Rodentia, Mammalia), 120 days old and weighing 462.33 ± 33.72 g randomly allocated into two main groups: the control group (C) and the experiment one (E). Each group was divided again into three groups of ten animals, according to the measurement time, and constituted the sub-groups C 3, C 7, C 14, E 3, E 7, and E 14.

The animals were maintained in the Surgical Technique and Experimental Surgery Laboratory with relative humidity proper to the environment and controlled temperature (20 ± 2°C), in a light-dark cycle of 12 h, and received water and feed (standard for the species) *ad libitum*.

### Study design

The sedated animals were weighed three days before the intervention, so that the dose and volume of VPA and the volume of 0.9% sodium chloride solution to be administered could be calculated. At this time, they were identified.

The rats in the experimental group received 100 mg/kg of VPA^28^. It was administered once a day, by gavage. It began three days before the intervention, and it was maintained until the date scheduled for the measurement. The animals in the control group received an equivalent volume of 0.9% sodium chloride solution by the same method.

Anesthesia and analgesia were conducted by a veterinarian. Anesthesia was initiated with a pre-anesthetic intramuscular injection of ketamine hydrochloride 50 mg/kg combined with xylazine hydrochloride 2 mg/kg. Anesthetic induction was performed by inhalation with 1% isoflurane, and maintenance with the same drug at 1.5% under mask, associated with 100% oxygen.

For postoperative analgesia, tramadol hydrochloride (5 mg/kg) was administered intra-muscularly.

### Surgical procedure

The anesthetized rats had their ventral region shaved and were identified. This was followed by antisepsis with 1% chlorhexidine digluconate.

All the procedures were performed by a single surgeon. A median incision of approximately 5 cm was made in the ventral abdominal wall. This was followed by the synthesis, of a continuous running suture of 4 monofilament nylon.

After recovery from anesthesia and analgesia, the animals were returned to their cages, medicated daily, and observed.

To verify the results, the animals were anesthetized and euthanized by a veterinarian, according to the protocol described in the CONCEA Euthanasia Practice Guidelines, Resolution no. 37, of the Ministry of Science, Technology, Innovation and Communication, of February 15, 2018.

### Evaluation criteria

The presence or absence of wound dehiscence and secretions were verified.

The embedded material underwent 4-μm thick sections that were assembled on slides and stained with hematoxylin-eosin (HE). For the histological analysis of the scar and the intensity and quality of the inflammatory reaction to be recognized, the methodology of Vizzotto Junior et al.^29^ was used. The type and number of predominant cells in the inflammatory reaction (poly and monomorphonuclear) were evaluated, along with the presence of interstitial edema and vascular congestion, and the degree of formation of granulation tissue and fibrosis. The indices were added up so that each group. The final score allowed to classify the inflammatory process in acute, sub-acute and chronic.

Another slide containing three sections was mounted and stained using the picrosirius–red F3BA technique. In this staining, with the examination performed under polarized light, the thicker and birefringent collagen fibers present a color ranging from yellow to red and represent type I collagen. The thinner and dispersed fibers, weakly birefringent, have a greenish color and represent type III collagen^30^,^31^. The images were captured by a Sony CCD101^®^ camera. The images were analyzed using the MediaCybernetics application Image-Plus^®^ 4.5 for Windows. Ten fields were analyzed with 400x magnification along the scar line. The field area that was read totaled 142.901 μm. In each field, the percentage of the area occupied by yellow and red (collagen I) and green (collagen III) fibers was calculated. Considering that the other types of collagen are represented by very small fractions, for practical purposes, the sum of collagens I and III was considered to be the total collagen of the scar. From the ten fields read, an average was obtained, which was considered for each animal.

For the immunohistochemical evaluations, the tissue array or microarray technique was used. The streptavidin-biotin-peroxidase technique was used, as described by Hsu et al.^32^. Anti-LCA monoclonal antibodies, anti-CD34 (for the identification of endothelial cells), anti-collagen I, anti-collagen III, and anti-alpha-smooth muscle (anti-ASM) were used to identify the myofibroblasts.

For all the parameters, ten fields were read, and an average was obtained. The readings were made using the MediaCybernetics application Image-Plus^®^ 4.5 for Windows^®^ on a microcomputer after the images were captured.

### Statistical analysis

The results of quantitative variables were presented by mean, standard deviation, median, minimum, maximum, and interquartile range (IQR). Categorical variables were presented by frequency and percentage. To compare the control and experiment groups, regarding quantitative variables, Student’s t-test for independent samples or the non-parametric Mann-Whitney’s test was used. Days of sacrifice (three, seven and 14 days) were compared using the one-way analysis of variance (ANOVA) model, the post-hoc Bonferroni test or the non-parametric Kruskal-Wallis test and Dunn’s post-hoc test. For the analysis of categorical variables, Fisher’s exact test was used. Values of p<0.05 indicated statistical significance. For multiple comparisons of sacrifice days, p-values were corrected by Bonferroni test. The data were analyzed using the IBM SPSS Statistics (Armonk, NY: IBM Corp.) computer program, v.28.

## Results

No secretion was found in the wounds at any time, and similar signs of epithelialization were seen at the three evaluation moments.

The inflammatory reaction, on the third day, showed acute and sub-acute reactions with no significant difference between the groups. At the seventh and the 14^th^ day, in the histological sections of the wounds of the control group, the chronic condition predominated, while in the experimental group the subacute condition predominated (seven days p = 0.006, and 14 days p = 0.020) (Table 1).

**Table 1:** Inflammatory reaction quality.

Inflammatory reaction	3 days			7 days			14 days	
	Cont.	Exp.		Cont.	Exp.		Cont.	Exp.
Acute/sub-acute	11	10		2	9		3	9
	100%	100%		20%	90%		30%	90%
Cronic	0	0		8	1		7	1
	0%	0%		80%	10%		70%	10%
Total	11	10		10	10		10	10
p* (Cont. × Exp.)	1			0.006			0.020	

* Fisher’s exact test, p<0.05; Cont.: control group; Exp.: experimental group.

The number of leukocytes stained by anti-LCA was higher in the experimental group on the third day (p = 0.036). However, there was no difference in the counts performed at seven days (p = 0.280) and 14 days (p = 0.529) (Table 2).

**Table 2:** Mean number of leukocytes in 10 fields (marked by anti-LCA).

Variable	Day	Group	N	Average	Standard deviation	Median	Minimum	Maximum	AIQ	p* (Cont. × Exp.)
LCA	Day 3	Cont.	11	26.27	9.49	25	10	50	8	0.036
		Exp.	10	36	15.78	30	25	70	10	
	Day 7	Cont.	10	14.70	6.31	15	5	25	5	0.280
		Exp.	10	19	8.10	25	5	25	15	
	Day 14	Cont.	10	5.10	1.52	5	3	8	2	0.529
		Exp.	10	5.20	2.49	5	3	12	1	
p**		Cont.		d3 × d7 × d14: p<0.001	d3 × d7: p=0.142	d3 × d14: p<0.001	d7 × d14: p=0.026			
		Exp.		d3 × d7 × d14: p<0.001	d3 × d7: p=0.070	d3 × d14: p<0.001	d7 × d14: p=0.033			

* Non-parametric Mann-Whitney’s test (LCA) or Student’s t-test for independent samples;

** non-parametric Kruskal-Wallis test (LCA); AIQ: interquantum rage; Cont.: control group; Exp.: experimental group.

The vessel count, marked by anti-CD34, in 10 fields, showed, at the three times studied, a significantly lower mean in the experimental group (Table 3).

**Table 3:** Mean number of vessels in 10 fields (marked by anti-CD34).

Variable	Day	Group	N	Average	Standard deviation	Median	Minimum	Maximum	AIQ	p* (Cont. × Exp.)
CD34	Day 3	Cont.	11	15.45	2.94	15	11	20	6	0.002
		Exp.	10	10.40	3.44	11.50	5	15	6	
	Day 7	Cont.	10	15.50	2.88	15	12	20	5	<0.001
		Exp.	10	10.50	2.22	10.50	7	15	3	
	Day 14	Cont.	10	8.50	2.01	8.50	6	12	3	0.027
		Exp.	10	6.10	2.42	6	3	10	4	
p**		Cont.		d3 × d7 × d14: p<0.001	d3 × d7: p=1	d3 × d14: p<0.001	d7 × d14: p<0.001			
		Exp.		d3 × d7 × d14: p=0.001	d3 × d7: p=1	d3 × d14: p=0.005	d7 × d14: p=0.004			

* Student’s test for independent sample; p<0.05;

** one-way analysis of variance (ANOVA) test (CD34); p<0.05; AIQ: interquantum rage; Cont.: control group; Exp.: experimental group.

The identification of myofibroblasts made it possible to verify that they were rare in both groups on the third day. On the seventh day, they were present in moderate amounts in all the cuts that were evaluated, which also occurred on the fourteenth day (the third day → p = 0.111, the seventh day → p = 0.111, and the 14^th^ day → p = 0.370).

Collagen analysis by immunohistochemistry made it possible to recognize, at the three times in question, a greater amount of collagen I in the histological sections of the skins of the control group (Table 4) and a greater amount of collagen III, on all three occasions, in the experiment group (Table 5).

**Table 4:** Average percentage of collagen I found in the three times, studied identified by immunohistochemistry.

Day	Group	n	Average	Standard deviation	Median	Mínimum	Maximum	AIQ	p* (Cont. × Exp.)
Day 3	Cont.	11	15	5	15	10	20	10	<0.001
	Exp.	10	4.50	3.69	5	0	10	5	
Day 7	Cont.	10	56	5.16	60	50	60	10	<0.001
	Exp.	10	17.50	10.07	12.50	5	30	20	
Day 14	Contr.	10	98.50	2.42	100	95	100	5	<0.001
	Exp.	10	81.50	7.47	82.50	70	90	15	
p**	Cont.		d3 × d7 × d14: p<0.001	d3 × d7: p=0.022	d3 × d14: p<0.001	d7 × d14: p=0.038			
	Exp.		d3 × d7 × d14: p<0.001	d3 × d7: p=0.100	d3 × d14: p<0.001	d7 × d14: p=0.016			

* Non-parametric Mann-Whitney’s test (day 7 and day 14) or Student’s t-test for independent samples (day 3); p<0.05;

** non-parametric Kruskal-Wallis test; p<0.05; AIQ: interquantum rage; Cont.: control group; Exp.: experimental group.

**Table 5:** Average percentage of collagen III found in the three times, studied identified by immunohistochemistry.

Day	Group	n	Average	Standard deviation	Median	Mínimum	Maximum	AIQ	p* (Cont. × Exp.)
Day 3	Cont.	11	85	5	85	80	90	10	<0.001
	Exp.	10	95.50	3.69	95	90	100	5	
Day 7	Cont.	10	44	5.16	40	40	50	10	<0.001
	Exp.	10	82.50	10.07	87.50	70	95	20	
Day 14	Cont.	10	1.50	2.42	0	0	5	5	<0.001
	Exp.	10	18.50	7.47	17.50	10	30	15	
p**	Cont.		d3 × d7 × d14: p<0.001	d3 × d7: p=0.022	d3 × d14: p<0.001	d7 × d14: p=0.038			
	Exp.		d3 × d7 × d14: p<0.001	d3 × d7: p=0.100	d3 × d14: p<0.001	d7 × d14: p=0.016			

* Non-parametric Mann-Whitney’s test (day 7 and day 14) or Student’s t-test for independent samples (day 3); p<0.05;

** non-parametric Kruskal-Wallis test; p<0.05; AIQ: interquantum rage; Cont.: control group; Exp.: experimental group.

Collagen analysis by means of picrosirius-red F3BA, after the areas were evaluated and the averages obtained, showed a lower amount of total collagen in the experimental group, at the three times of evaluation. However, there was a gain in both groups as the process progressed (Table 6).

**Table 6:** Average area occupied by collagen, using picrosirius-red F3BA.

Day	Group	N	Average	Standard deviation	Median	Minimum	Maximum	AIQ	p* (Cont. × Exp.)
Day 3	Cont.	11	15.31	2.58	15.85	11.06	19.73	4.07	0.001
	Exp.	10	11.15	2.41	10.32	8.05	15.51	3.44	
Day 7	Cont.	10	19.58	3.08	18.68	15.72	25.62	5.16	0.004
	Exp.	10	15.03	3.07	15.91	9.39	18.72	4.75	
Day 14	Cont.	9	27.47	4.32	27.45	22.84	34.15	6.40	<0.001
	Exp.	10	19.39	2.58	19.03	15.87	23.22	3.57	
	Cont.		d3 × d7 × d14 p<0.001	d3 × d7: p=0.021	d3 × d14: p<0.001	d7 × d14: p<0.001			
	Exp.		d3 × d7 × d14 p<0.001	d3 × d7: p=0.010	d3 x d14: p<0.001	d7 × d14: p=0.004			

* Student’s t-test for independent sample; p<0.05;

**one-way analysis of variance (ANOVA) test and Bonferroni *post-hoc* test, p<0.05; AIQ: interquantum rage; Cont.: control group; Exp.: experimental group.

The percentage of area occupied by collagen I was also higher in the control group at the three times (Table 7).

**Table 7:** Average area occupied by collagen I, using picrosirius-red F3BA.

Day	Group	N	Average	Standard deviation	Median	Minimum	Maximum	AIQ	p* (Cont. × Exp.)
Day 3	Cont.	11	1.49	0.25	1.42	1.20	1.93	0.49	
	Exp.	10	0.83	0.21	0.80	0.62	1.31	0.26	<0.001
Day 7	Cont.	10	6.48	1.17	6.62	4.64	8.67	1.71	
	Exp.	10	2.75	0.81	2.77	1.53	4.29	1.12	<0.001
Day 14	Cont.	9	9.91	1.62	10.12	8.01	13.33	1.69	
	Exp.	10	6.53	0.83	6.37	5.07	7.91	1.18	<0.001
**	Cont.		d3 × d7 × d14 p<0.001	d3 × d7: p<0.001	d3 × d14: p<0.001	d7 × d14: p<0.001			
	Exp.		d3 × d7 × d14 p<0.001	d3 × d7: p<0.001	d3 × d14: p<0.001	d7 × d14: p<0.001			

* Student’s t-test for independent sample; p<0.05;

** one-way analysis of variance (ANOVA) test and Bonferroni *post-hoc* test, p<0.05; AIQ: interquantum rage; Cont.: control group; Exp.: experimental group.

The percentage of area occupied by collagen III was also higher in the control group at the three times. However, it was only significant on the third and on the 14^th^ day (Table 8).

**Table 8:** Average area occupied by collagen III, using picrosirius-red F3BA.

Day	Group	N	Average	Standard deviation	Median	Minimum	Maximum	AIQ	p* (Cont. × Exp.)
Day 3	Cont.	11	13.82	2.69	14.65	9.23	18.48	3.67	
	Exp.	10	10.32	2.31	9.65	7.39	14.59	3.63	0.005
Day 7	Cont.	10	13.10	2.50	13.04	9.13	16.95	4.46	
	Exp.	10	12.28	2.69	13.10	7.86	15.91	4.12	0.489
Day 14	Cont.	9	17.55	2.92	17.10	14.15	22.66	3.96	
	Exp.	10	12.87	2.15	12.35	10.37	15.91	3.75	<0.001
**	Cont.		d3 × d7 × d14 p=0.003	d3 × d7: p=1	d3 × d14: p=0.014	d7 × d14: p=0.004			
	Exp.		d3 × d7 × d14 p=0.061	-					

* Student’s t-test for independent sample; p<0.05;

** one-way analysis of variance (ANOVA) test and Bonferroni *post-hoc* test, p<0.05; AIQ: interquantum rage; Cont.: control group; Exp.: experimental group.

## Discussion

The healing of the skin wounds is a complex process that involves numerous and different cells. It comprises the inflammatory, proliferative and remodeling phases, which, although sequential, overlaps^33^,^34^.

Immediately after an injury, a clot forms and provides a matrix for the influx of inflammatory cells. The inflammatory phase is characterized by the migration of leukocytes to the wound, and their mainly function is to remove bacteria. Next, it comes the monocytes differentiate into macrophages, in addition to phagocytosis, exercising pro-inflammatory and anti-inflammatory functions, depending on the stage of the process. The deposition of the newly synthesized fibrin is followed by the formation of matrix and granulation tissue. The proliferative phase of wound healing is characterized by re-epithelialization, neovascularization, extracellular matrix, and collagen deposition^33^.

Although the histological aspect of the healing process is known, little is known about its molecular basis. It is highly likely that epigenetic regulation, including histone modifications and DNA methylation, plays a role in this process^35^. 

Within the cell, DNA is packaged in a hierarchical structure with the basic unit of a double-stranded DNA helix. Approximately 145 base pairs of DNA are wrapped around a hetero-octameric histone protein complex (H2A, H2B, H3, and H4), and together the DNA-histone complexes are called nucleosomes^36^.

Nucleosomes are intercalated along the DNA with short sequences between them, allowing for interactions between separate nucleosomes and the subsequent formation of chromatin strands. Chromatin can be compact (heterochromatin) or loose structure (euchromatin), which serves as a form of gene regulation, as loosely packed DNA is more accessible for protein binding and transcription^36^.

Types of histone modifiers include: acetylation, methylation, phosphorylation, and ubiquitination. Histone methylation and ubiquitination are associated with gene activation and repression, while histone acetylation is typically associated with gene activation^37^.

Macrophages are essential since the inflammatory response to the resolution of wound healing. Histone methyltransferases play an important role in regulating the transition of macrophages from a pro-inflammatory to an anti-inflammatory phenotype^34^.

Granulation tissue has a high proportion of collagen III. As the healing process progresses, the extracellular matrix is enriched with collagen I^38^.

Other components of the extracellular matrix include fibronectin, elastin, laminins, proteoglycans, hyaluronic acid, glycoproteins, and proteins that are primarily synthesized by fibroblasts, but epithelial, endothelial, and immune cells also contribute^39^.

In human keratinocyte culture, HDAC inhibitors were found to cause an accumulation of acetylated histones, with inhibition of keratinocyte growth. However, fibroblasts were not inhibited^40^. 

The effects can differ depending on which histone is being deacetylated. Class I histones, HDAC2 inhibits the expression of growth factors important for keratinocyte proliferation and wound healing, such as insulin growth factor I (IGF-I), the fibroblast growth factor 10 (FGF-10) and epidermal growth factor (EGF). Meanwhile, class III HDACs (Sirtuins) downregulate class I HDACs and therefore promote keratinocyte proliferation through increased NO synthesis by endothelial cells^41^.

It is recognized that VPA acts as a class I selective inhibitor of histone deacetylase enzymes, responsible for removing the methyl group from histones, thus exiting the S cell phase^21^. DNA remains hypermethylated, blocking the cell cycle^2^.

In the study described here, the inflammatory reaction was acute or subacute, in both groups, in the three-day evaluation. In the seven- and 14-day checks, it was predominantly of the chronic type in the control group and subacute in the experiment group, which was treated with VPA. An analysis of the cellularity showed that there were more leukocytes in the histological sections of the treated group on the third day and, although their numbers were greater on the other two occasions, the difference was not significant. This leads us to think that initially the inflammatory process was more intense in the treated group.

Contrary to the findings of the consulted authors^5^-^8^, who reported an increase in collagen types I and III, when immunohistochemical staining was used, there was less collagen I in the sections obtained from the treated animals and more type III collagen. This finding supports the hypothesis that the healing process of the animals treated with VPA was less evolved than in the control group. This was confirmed by analyzing sections treated with picrosirius-red F3BA under polarized light. It can be seen that there was more collagen in the scars of the skins of the control group, with a higher percentage of type I collagen.

Antiangiogenic effects of VPA were verified *in vitro* and *in vivo*. *In vitro*, it preferentially inhibited endothelial cell proliferation and reduced VEGF secretion and inhibited tube formation in the angiogenesis assay^10^. These anti-angiogenic effects are associated with the downregulation of pro-angiogenic genes, such as VEGF and/or endothelial nitric oxide synthase (eNOS) genes. Moreover, HDAC inhibitors induce hyperacetylation of HIF-1α, a pro-angiogenic transcription factor, which induces its degradation^11^.

*In vitro*, VPA was shown to inhibit eNOS, thus directly decreasing NO levels, which participate in endothelial proliferation, migration, and organization of angiogenesis^5^,^42^-^44^.

In this study, a three times lower number of vessels was observed in the treated group. This can be explained by the statements of Rössig et al.^42^, who claimed that VPA decreases cyclic guanylate-monophosphate and eNOS and as a consequence VEGF would remain low. Furthermore, Michaelis et al.^4^ reported that VPA directly inhibits angiogenesis by reducing the replication of endothelial cells.

The lower number of vessels may partly account for the lower collagen deposition, since this synthesis is oxygen dependent.

Despite alterations in the intensity of the inflammatory reaction, which constitutes the initial phase, lower collagen density and lower number of neovessels, there was no interference in the number of myofibroblasts, Brinkmann et al.^40^ had previously reported that VPA did not affect fibroblasts.

Changes were found at the microscopic level, although there was no macroscopic damage. It is important to recognize these changes so that when intervening in a cancer patient who has received or is receiving treatment associated with epigenetics, in this special situation VPA, care is redoubled, because, in addition to the damage to healing resulting from chemotherapy or even radiotherapy, the harm caused by epigenetic blockage is added.

## Conclusion

The administration of VPA led to a more intense inflammatory reaction, decreased angiogenesis and collagen deposition, especially type I collagen.
